# The Lag Structure and the General Effect of Ozone Exposure on Pediatric Respiratory Morbidity

**DOI:** 10.3390/ijerph8104013

**Published:** 2011-10-20

**Authors:** José Fraga, Anabela Botelho, Aida Sá, Margarida Costa, Márcia Quaresma

**Affiliations:** 1 Department of Pediatrics at Centro Hospitalar de Trás-os-Montes e Alto Douro, Avenida da Noruega-Lordelo, Vila Real 5000-508, Portugal; E-Mails: zefraga@iol.pt (J.F.); aida-sa@hotmail.com (A.S.); anampbc@gmail.com (M.C.); marciaquaresma@hotmail.com (M.Q.); 2 University of Minho and NIMA, Campus de Gualtar, Braga 4710-057, Portugal

**Keywords:** public health, respiratory morbidity, children, surface ozone, distributed lag, non-linear models, delayed effects

## Abstract

Up to now no study has investigated the lag structure of children’s respiratory morbidity due to surface ozone. In the present study, we investigate the lag structure and the general effect of surface ozone exposure on children and adolescents’ respiratory morbidity using data from a particularly well suited area in southern Europe to assess the health effects of surface ozone. The effects of surface ozone are estimated using the recently developed distributed lag non-linear models, allowing for a relatively long timescale, while controlling for weather effects, a range of other air pollutants, and long and short term patterns. The public health significance of the estimated effects is higher than has been previously reported in the literature, providing evidence contrary to the conjecture that the surface ozone-morbidity association is mainly due to short-term harvesting. In fact, our data analysis reveals that the effects of surface ozone at medium and long timescales (harvesting-resistant) are substantially larger than the effects at shorter timescales (harvesting-prone), a finding that is consistent with all children and adolescents being affected by high surface ozone concentrations, and not just the very frail.

## 1. Introduction

Surface ozone is a pollutant of growing concern in Europe [1], and children represent the largest subgroup of the population susceptible to the adverse health effects of surface ozone concentrations [2], particularly in terms of respiratory diseases [3,4]. However, there are as yet relatively few epidemiological time-series studies assessing the effects of current European surface ozone levels on children and adolescents’ respiratory morbidity. Those that exist have been mainly conducted in large urban areas, and the estimated effects tend to be relatively small in magnitude. For example, a recent meta-analysis of European studies provided a summary relative risk of 0.999 for respiratory admissions in children aged 0–14 years per 10 μg/m^3^ surface ozone increase [5]. One factor that may account for this finding is exposure misclassification (*i.e.*, poor correlation between the commonly used surface ozone levels measured at fixed sites and personal exposure), an occurrence that tends to cause an underestimation bias in the health effect estimates, particularly in urban areas [1]. In addition, the strong inverse correlation between surface ozone and fine particles from traffic sources in many cities may confound or conceal the real effects of surface ozone, or even explain the protective effect of surface ozone that has been found in some European studies [2], particularly during the winter. Thus, it has been suggested that ambient measurements in warmer places, where people spend more time outdoors, recorded at rural background stations not affected by local traffic pollutants may better represent the actual average population exposure, thereby avoiding the aforementioned confounding factors [6–9].

**Figure 1:**
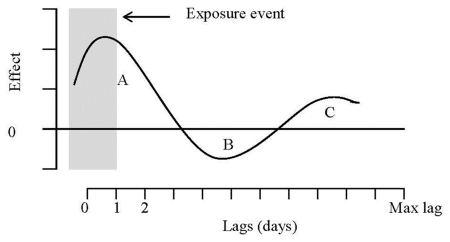
Lag structure corresponding to a morbidity displacement effect.

Moreover, the existing studies tend to assess the health effects of changes in surface ozone levels using relatively short timescales [10,11]. If all extra emergency room visits or hospital admissions in days following higher surface ozone levels are occurring only a few days early among children already near to acute health events (harvesting, or morbidity displacement), we would expect little association between exposure to higher surface ozone levels and morbidity counts a few days after [12]. Statistical aspects associated with a morbidity/mortality displacement effect have been discussed by several authors [13], and a conceptual framework for this effect is presented in Figure 1. Area A corresponds to the sum of positive effects on morbidity for highly vulnerable individuals whose illness is being advanced a few days following a high episode of surface ozone. Exposure on that earlier day (which produced the short-term morbidity advancement) is then negatively associated with morbidity in subsequent days, when fewer individuals will become ill than otherwise because some of the illnesses have been shifted forward a few days or weeks. This corresponds to the so-called “harvesting” effect, and is captured by area B (sum of negative effects) in the figure. As pointed out by Zanobetti *et al*. [13], however, if the increased risk of illness following a high episode of surface ozone dies out slowly over time, or if there is increased recruitment (by moving people from moderate to severe illness) into the risk pool of vulnerable individuals due to surface ozone, and this occurs at a slower pace than the effects illustrated in period A, then a period C subsequent to the harvesting period may also be observed where the effects on morbidity are once again positive. This effect is thought to be important in conceptual models of the effects of air pollution on morbidity counts (particularly on hospital admissions) showing how these health outcomes can be brought forward by a relatively short period of time, as well as events being added that would not have happened except for air pollution [14]. The net impact is given by the sum of these areas, and depends on the relative size of each effect.

Up to now, however, no study has investigated the lag structure of children’s respiratory morbidity due to surface ozone, and only one study has so far analyzed the mortality displacement due to surface ozone using data for 48 cities in the United States [15], and another study using data for 21 cities in Europe [16]. The authors using the US data show that the sum of the surface ozone effects distributed over three weeks after exposure is larger than the effect estimated using a single day of surface ozone exposure for all causes and cause-specific mortality (including cardiovascular and respiratory mortality). Thus, this study indicates that risk assessments using the same or next day after exposure are likely to underestimate, rather than overestimate, the public health impact. This finding is replicated in the study using the European data, but only with respect to respiratory mortality. Whether the lag structure of children’s respiratory morbidity due to surface ozone causes similar underestimation problems in studies using short timescales is an open question, but the use of longer timescales, presumably resistant to displacement, is warranted in light of this evidence, along with the findings previously reported in the literature that children tend to express symptoms due to surface ozone less ready than adults do [17,18].

In the present study, we investigate the lag structure and the general effect of surface ozone exposure on children and adolescents’ respiratory morbidity expressed in terms of emergency room visits and admissions due to lower airways diseases in a district hospital located in a northeast rural area of Portugal in southern Europe. This is a particularly well suited area to assess the health effects of surface ozone given that concentrations in southern Europe are higher than in northern Europe and are higher in rural than in urban areas [1,7]. Ambient ozone measurements in this area may also better represent the population exposure across Europe in the coming decade as surface ozone concentrations are expected to increase and regional differences in exposure levels to diminish, due to increasing background levels (as currently captured by those measured at rural background stations) and reduced surface ozone depletion in urban areas [1].

## 2. Methods

We use daily air quality, meteorological, and hospitalization data for 2004 through 2008 for the rural district of Vila Real in Portugal. The source of the air pollution data is the Portuguese Environmental Agency (www.qualar.org), and includes hourly measurements (in μg/m^3^) taken at the relevant rural background station of Lamas d’Olo, located in the northeast of Portugal, where surface ozone (O_3_) exceedances are very frequent. At this site, pollutants like ozone, sulphur dioxide (SO_2_), nitrogen dioxide (NO_2_), particulate matter with aerodynamic diameter less than 2.5 μm (PM_2.5_), and particulate matter with aerodynamic diameter smaller than 10 μm (PM_10_) have been monitored since 2004, with hourly acquisition efficiency above 80% [19]. On average, 30% of the total alert threshold exceedances observed by the national monitoring network are registered at this station, reaching more than 40% (and more than 80% of the information threshold exceedances) in 2005 when the highest surface ozone concentrations in Europe where registered at this station [19,20]. The annual average surface ozone concentration at this site is about 100 μg/m^3^, a value particularly high when compared to the 40–90 μg/m^3^ average levels detected over the midlatitudes of the Northern Hemisphere [19,21].

Concerning the monthly distribution of surface ozone concentrations, the highest values are observed in the summer months (June, July, August and September), but, as already detected by several authors in other different parts of the world, surface ozone peaks are also observed in the spring, namely in April [19,22]. As has been previously proposed [2], we restricted our evaluation period to the April–September months. In addition to exclude seasonal influenza activity, these are also the months when children and adolescents spend more time outdoors, and ambient ozone measurements better reflect personal exposure [4,9].

Although the levels of other air pollutants tend to be relatively low in the study area, the hourly measurements of SO_2_, NO_2_, PM_2.5_, and PM_10_ were also collected and controlled for in the analysis as they have been previously identified as potential effect modifiers of surface ozone in studies of co-pollutant health effects on children and adolescents [23]. Along with surface ozone, all these hourly measurements were collapsed to daily average values. Although different epidemiological studies have used different indicators of surface ozone exposure (maximum daily 1- or 8-hour average are commonly used indicators), a recent systematic examination of all three indicators conducted by Triche *et al*. [24] indicates that that the 24-hour average is more relevant to respiratory symptoms than either of the other two indicators, despite the fact that all three indicators are highly correlated [1]. In order to ensure an equally-spaced and ordered series of the same season for each year, missing data for the air pollutants were imputed following the procedure proposed by Zanobetti *et al*. [13] (the percentage of imputed values is 8.6%). Meteorological measurements were also collected, and controlled for in the analysis, to account for the possible confounding effect of weather on the relationship between surface ozone and morbidity. The weather measurements are provided by the National Meteorological Service for the study area (Vila Real district), and include daily values for average temperature (in °C), and relative humidity.

The study group consists of all children and adolescents under the age of 18 who resorted to the pediatric emergency room service of the hospital center known as Centro Hospitalar de Trás-os-Montes e Alto Douro (CHTMAD), which serves as the health reference center for the entire district, during the period between 1 April–30 September for the years 2004–2008, and were diagnosed with lower respiratory tract diseases (*i.e.*, any infectious or inflammatory disease of the lower airways, including pneumonia, bronchiolitis and asthma exacerbation; all upper airway respiratory disease and trauma pathology were excluded). The daily counts of hospital emergency room visits and daily counts of hospital admissions under the referred conditions were obtained through consultation and analysis of the emergency charts and clinical processes, on paper or digital format, using the Medical IT Support System (SAM) of the hospital center during the selected period.

### Statistical Methods

Estimation of the effects of environmental factors on health outcomes must take into account: (i) the nature of the dependent (response) variable; (ii) the potential nonlinear relationship between the dependent variable and the covariates (predictors); and (iii) the potential delayed effects of the covariates on the dependent variable. In this study, the outcomes of interest are daily counts of hospital emergency room visits and daily counts of hospital admissions, both of which can only take values limited to the nonnegative integers. This suggests that a Poisson process generates the data. However, estimation of a Poisson regression model is only appropriate if the data is equidispersed (the variance is equal to the mean function), a feature that is commonly violated in count data. When the data is overdispersed (variance larger than the mean), as in the current application, statistical inference from the Poisson regression is incorrect as it fails to account for the excess variation around the model’s fitted values [25]. This issue can be addressed through the estimation of a so-called quasi-Poisson model which basically consists in estimating an additional “dispersion parameter” from the data, and using it to adjust inference for overdispersion [25].

In their standard formulation, these models assume a linear relationship between the dependent variable and the predictors (or some other pre-defined mathematical function, such as a polynomial, *etc*.). In environmental epidemiologic studies, however, the relationship between the response variable and some of the predictors is expected to be nonlinear in ways that cannot be easily defined a priori. The generalized additive models (GAM) proposed by Hastie and Tibshirani [26–28] specify the predictor as an additive function of a transformation of the original predictors (smooth functions), and the related parameters can be estimated within the generalized linear models’ framework using a quasi-Poisson family of distributions. In addition to possible nonlinearities in the dimension of the predictors, environmental epidemiologic studies must take into account the possible time lags between changes in the predictors and their effects on health. The so-called distributed lag models (DLM) with Almon lag schemes [29] are commonly used in environmental epidemiologic studies to take the temporal dimension of the predictors into account.

These two types of models, the GAM and the DLM, can be combined giving rise to generalized additive distributed lag models (GADLM) as proposed by Zanobetti *et al*. [13]. In their formulation, the response variable (assumed to follow a Poisson distribution) is modeled as an additive function of smoothed predictors plus their polynomial distributed lagged effects. Although incorporating both nonlinearities in the dimension of the predictors and their temporal dimension, this approach keeps these effects separated. Recently, Gasparrini *et al*. [30] developed the so-called distributed lag non-linear models (DLNM) that allow nonlinear dependencies and lagged effects to be modeled simultaneously in quite flexible ways. The mathematical formulation of DLNM is quite complex [30,31], but it relies on a simple concept: a known set of transformations (basis functions) of the original predictor are independently set to describe the shape of the relationship in each dimension, and these functions are then combined to generate a cross-basis function which is a bi-dimensional space of functions describing simultaneously the shape of the relationship along the space of the predictor and along its temporal dimension.

In this study, we apply the DLNM framework recently implemented within the statistical software R [31] to assess the effects of daily surface ozone measurements on daily counts of hospital emergency room visits and daily counts of hospital admissions, while controlling for weather effects, a range of other air pollutants, and long and short term patterns. The relationship with surface ozone is modeled through a natural cubic spline with 4 degrees of freedom and boundary knots located at the range of the observed values. The relationship in the space of the other air pollutants and weather variables is modeled through a natural cubic spline of 4th order. In all cases, the lagged effect is specified up to 30 days of lag with a 5th degree polynomial function In addition, indicator variables for year, month of the year, season of the month (spring and summer), an interaction term between month and season, and day of the week, are included in the model to control for short-term confounders and seasonality. The specification for the degrees of freedom in each dimension is chosen so as to minimize the quasi-Akaike Information Criterion [30]. A maximum lag of 30 days is allowed in order to examine the lag structure of children’s respiratory morbidity due to surface ozone.

## 3. Results and Discussion

Table 1 presents descriptive statistics of daily hospital emergency room visits (ERV), hospital admissions (HA), air pollutants and weather variables, from 2004 through 2008. During the period between 1 April and 30 September for the years 2004–2008, there were 1952 ERV, and 350 HA, due to lower respiratory tract diseases.

The maximum number of daily hospital ERV (14), and the maximum number of daily HA (6) were both registered in year 2005. This was also the year that registered the highest number of days in which average daily surface ozone concentrations (average concentration =126.12 μg/m^3^, varying between 65.79 μg/m^3^ and 235.73 μg/m^3^) exceeded the current EU target value for surface ozone concentrations (120 μg/m^3^ for 8 h running average; Directive 2008/50/EC). In fact, this target value was exceeded 88 times in 2005, corresponding to 48% of the 183 observed values. Overall, the target value was exceeded in 23% of the 915 days under observation. The lowest number of exceedances was registered in 2007, corresponding to 8% of the 183 observed values for that year. The lowest average daily surface ozone concentrations also occurred in 2007 (average concentration =92.10 μg/m^3^, varying between 39.38 μg/m^3^ and 150.46 μg/m^3^). In fact, as detected by other authors [32], surface ozone levels during the 2007 summer were among the lowest in the past decade in Portugal, and, accordingly, the number of exceedances in Europe was also lower in that summer than in any of the last 10 summers. The levels of the other pollutants tend to be low. The average daily concentration of PM_10_ for the 915 days under observation is 21.74 μg/m^3^. This value is about half of the daily limit value adopted in Europe for this particulate matter, which is 40 μg/m^3^. However, the limit value was exceeded in 7% of the 915 days. Again, the highest number of exceedances occurred in 2005 (28 times), corresponding to 15% of the 183 daily values. The average daily concentration of PM_2.5_ varied between 3.75 μg/m^3^ in 2008 and 15.29 μg/m^3^ in 2005 (average concentration =10.66 μg/m^3^), never exceeding the yearly limit value of 25 μg/m^3^. Similarly, the concentrations of SO_2_ and NO_2_ were very low, never exceeding their respective limit values (daily limit value of 125 μg/m^3^ for SO_2_, and yearly limit value of 40 μg/m^3^ for NO_2_), indicating very low traffic influence.

Pearson correlation coefficients between the considered air pollutants and meteorological variables are presented in Table 2. The results show that surface ozone is positively correlated with PM_2.5_, PM_10_, and NO_2_ at the 5% significance level; the correlation of surface ozone and SO_2_ is small in magnitude and statistically insignificant. All the pollutants are substantially correlated with the weather variables at the 5% significance level: they are positively correlated with temperature and negatively correlated with humidity.

As explained above, the effect of surface ozone on daily counts of hospital ERV and daily counts of HA was estimated within the DLNM framework using a generalized linear model (GLM) with quasi-Poisson family to account for overdispersion (the estimated dispersion parameter is 1.33 and 1.16 in the ERV model and in the HA model, respectively).

**Figure 2:**
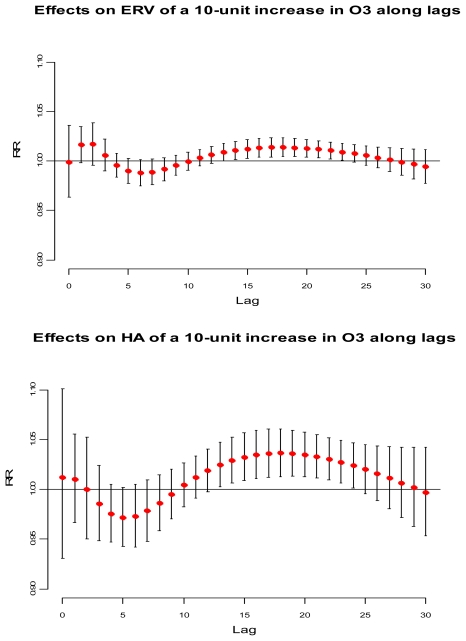
Effects of RR for ERV (top panel) and HA (bottom panel) on a 10 μg/m^3^ increase in surface ozone concentration level along lags. Reference at 120 μg/m^3^.

Figure 2 illustrates the lag-specific effects for a 10 μg/m^3^ increase in surface ozone concentrations compared with a reference value of 120 μg/m^3^, the current EU target value for surface ozone concentrations. The top panel illustrates the relative risk (RR) concerning ERV along with 95% confidence interval, and the bottom panel illustrates the RR concerning HA. Importantly, in each case the figure shows the basic features of the lag structure postulated in Figure 1.

A short-term advancement is observed for ERV at lags 0–3, and at lags 0–2 for HA. A harvesting period is also observed at lags 4–10 and at lags 3–9 for ERV and HA, respectively. In each case, the harvesting period is followed by a long period where positive effects tend to persist, suggesting long-term effects. The use of different health outcomes, different metrics for surface ozone exposure, different co-pollutants, and different number of lags may account for some of the inconsistency in findings in studies conducted both in Europe and outside Europe (reviewed by the WHO [2]), and make the comparison of our results with the existing literature somewhat difficult. However, the association between a 10 μg/m^3^ increase in surface ozone concentrations and HA at lags 0–2 found in the current study (RR = 1.02) equals the comparable RR found in the meta-analysis of European studies using daily counts of hospital admissions for total respiratory conditions among children under the age of 14 in Rome (RR = 1.02 at lag 1; 95% CI: 1.00–1.04) [5,10]. This result is also consistent with comparable estimates provided by the meta-analysis of European studies using data from London (RR = 0.99 at lag 0, 95% CI: 0.99–1.00), and West Midlands (RR = 0.99 at lags 0–1; 95% CI: 0.98–0.99) in the United Kingdom [5,33,34].

Table 3 presents the overall estimated RR (summing up the contributions for the 30 days of lag, *i.e.*, A+B+C) computed for a 10 μg/m^3^ increase in surface ozone concentrations compared with a reference value of 120 μg/m^3^. All the RR proved positive, and were statistically significant (p < 0.05). A decomposition of the surface ozone effect in three parts A, B, and C as in Figures 1 and 2 are also presented in Table 3. The overall RR for ERV is 1.15 (95% CI: 1.01–1.29). As expected, the severity of the health effects caused by increased surface ozone levels is greater for individuals that require hospital admission than for those individuals looking for emergency care. The overall RR for HA is 1.42 (95% CI: 1.02–1.97). In each case, long-term effects play a major role in the overall results. In fact, short-term displacement exerts a small effect both on ERV and on HA. On the other hand, short-term harvesting is substantial to both outcomes, representing the depletion of the risk pool.

It is important to notice that the sum of periods A+B underestimates the public health significance of average daily surface ozone concentrations. Had the analysis failed to allow for a relatively long distributed lag, no statistically significant association would have been found between surface ozone levels and morbidity. Period C, corresponding to the sum of the last 20 days for ERV and to the sum of the last 21 days for HA, is responsible for the high relative risks observed. This may be due to an increase of the children at risk [10,11], or to harmful effects of surface ozone exposure that persist for an extended time [8,35], or to both.

## 4. Conclusions

In the present study, we investigate the lag structure and the general effect of surface ozone exposure on children and adolescents’ respiratory morbidity expressed in terms of daily ERV and HA due to lower airways diseases in a district hospital located in a northeast rural area of Portugal in southern Europe. The effects of surface ozone are estimated using the recently developed distributed lag non-linear models allowing for a relatively long timescale, while controlling for weather effects, a range of other air pollutants, and long and short term patterns. The effect of surface ozone associated with a 10 μg/m^3^ increase above the European reference value on both health end-points is found distributed over an extended time, with a cumulative relative risk of 1.15 (95% CI: 1.01–1.29) for ERV, and a cumulative relative risk of 1.42 (95% CI: 1.02–1.97) for HA. The public health significance of these effects is higher than has been previously reported in the literature, providing evidence contrary to the conjecture that the surface ozone-morbidity association is mainly due to short-term harvesting. In fact, our data analysis reveals that the effects of surface ozone at medium and long timescales (harvesting-resistant) are substantially larger than the effects at shorter timescales (harvesting-prone), a finding that is consistent with all children and adolescents being affected by high surface ozone concentrations, and not just the very frail.

## Figures and Tables

**Table 1:** Descriptive statistics of daily values of variables used in the analysis.

Variables	Mean	SD	Min	Max	N
*Lower respiratory diseases*					
ERV	2.13	2.00	0.00	14.00	915
HA	0.38	0.71	0.00	6.00	915
*Pollutants*					
O_3_ (μg/m^3^)	104.30	27.10	39.38	235.73	915
PM_2.5_ (μg/m^3^)	10.66	9.35	0.50	97.21	915
PM_10_ (μg/m^3^)	21.74	14.81	0.40	184.96	915
SO_2_ (μg/m^3^)	2.61	3.00	0.00	20.04	915
NO_2_ (μg/m^3^)	2.74	2.08	0.00	22.88	915
*Weather*					
Temperature (°C)	18.35	4.88	5.00	30.80	915
Relative humidity (%)	63.85	15.41	23.00	96.00	915

**Table 2:** Matrix of Pearson correlation coefficients between air pollutants and weather variables.

Variables	O_3_	PM_2.5_	PM_10_	SO_2_	NO_2_	Temperaure
O_3_	1.000					
PM_2.5_	0.4688*	1.000				
PM_10_	0.4457*	0.7888*	1.000			
SO_2_	0.0023	0.0288	0.1489*	1.000		
NO_2_	0.2460*	0.4878*	0.5088*	0.3636*	1.000	
Temperature	0.3575*	0.4056*	0.4315*	0.1834*	0.3300*	1.000
Rel. humidity	−0.4458*	−0.2915*	−0.3106*	−0.1676*	−0.1774*	−0.5282 *

* Statistically significant at p-value ≤ 0.05.

**Table 3:** Relative risk estimates and 95% confidence intervals.

Periods	Emergency Room Visits			Hospital Admissions		
	
	RR	95% CI		RR	95% CI	
A	1.04	0.98	1.10	1.02	0.90	1.16
B	0.95	0.92	0.99	0.87	0.79	0.96
C	1.16	1.13	1.19	1.59	1.42	1.77
A+B	0.99	0.90	1.09	0.89	0.72	1.11
A+B+C	1.15	1.01	1.29	1.42	1.02	1.97
